# Validation of Suitable Reference Genes for Assessing Gene Expression of MicroRNAs in *Lonicera japonica*

**DOI:** 10.3389/fpls.2016.01101

**Published:** 2016-07-26

**Authors:** Yaolong Wang, Juan Liu, Xumin Wang, Shuang Liu, Guoliang Wang, Junhui Zhou, Yuan Yuan, Tiying Chen, Chao Jiang, Liangping Zha, Luqi Huang

**Affiliations:** ^1^State Key Laboratory Breeding Base of Dao-di Herbs, National Resource Center for Chinese Materia Medica, China Academy of Chinese Medical SciencesBeijing, China; ^2^Beijing Institute of Genomics, Chinese Academy of SciencesBeijing, China

**Keywords:** *Lonicera japonica*, microRNAs, qRT-PCR, reference gene, normalization, validation, different varieties

## Abstract

MicroRNAs (miRNAs), which play crucial regulatory roles in plant secondary metabolism and responses to the environment, could be developed as promising biomarkers for different varieties and production areas of herbal medicines. However, limited information is available for miRNAs from *Lonicera japonica*, which is widely used in East Asian countries owing to various pharmaceutically active secondary metabolites. Selection of suitable reference genes for quantification of target miRNA expression through quantitative real-time (qRT)-PCR is important for elucidating the molecular mechanisms of secondary metabolic regulation in different tissues and varieties of *L. japonica*. For precise normalization of gene expression data in *L. japonica*, 16 candidate miRNAs were examined in three tissues, as well as 21 cultivated varieties collected from 16 production areas, using GeNorm, NormFinder, and RefFinder algorithms. Our results revealed combination of u534122 and u3868172 as the best reference genes across all samples. Their specificity was confirmed by detecting the cycling threshold (*C*_t_) value ranges in different varieties of *L. japonica* collected from diverse production areas, suggesting the use of these two reference miRNAs is sufficient for accurate transcript normalization with different tissues, varieties, and production areas. To our knowledge, this is the first report on validation of reference miRNAs in honeysuckle (*Lonicera* spp.). Restuls from this study can further facilitate discovery of functional regulatory miRNAs in different varieties of *L. japonica*.

## Introduction

*Lonicera japonica* Thunb. (FLJ), commonly known as Japanese Honeysuckle or “Ren Dong,” has been widely used in traditional medicine in East Asian countries for thousands of years (Jiang et al., 2009). Its dry flower buds, leaves, and stems may be used to prevent and to treat a variety of illnesses, such as hand-foot-and-mouth disease, H1N1 influenza, pancreatic cancer, and some severe acute respiratory syndromes (Kwak et al., 2005; Kang et al., 2007; Lin et al., 2016). However, different parts of this plant have different medicinal properties: the flower buds have anti-inflammatory and anticancer properties, the leaves have antioxidant and tyrosinase-inhibitory activities, and the stems have xanthine oxidase-inhibitory and nitrite-scavenging activities (Vukovic et al., 2012). Several secondary metabolites have been identified and isolated from this plant, including biflavonoids, quercetin, phenolic acids, and dicaffeoylquinic acid (Fu et al., 2013), which are thought to be responsible for these various biological and pharmacological activities (Liu et al., 2016; Zhang B. et al., 2016). Quantitative differences, comparing the chemical composition of different parts of *L. japonica*, have been investigated: (1) the percentage of oxygenated monoterpenes and chlorogenic acid decreased from flower > leaf > stem; (2) the level of hexadecanoic acid increased from flower < leaf < stem; and (3) the content of luteoloside decreased from leaf > flower > stem (Vukovic et al., 2012; He, 2013; Jiang et al., 2013). In contrast, the content of active compounds also differs significantly among the varieties and production areas of *L. japonica*, leading to the different pharmacological activities and medicinal qualities (Yuan et al., 2012; Shi et al., 2016; Wu et al., 2016). *L. japonica* Thunb. var. *chinensis* (Watts; rFLJ), a Chinese endemic variety, has different active compound contents from those of FLJ (Yuan et al., 2012; **Supplementary Figure S1**). Both varieties of *L. japonica* are widely planted in different production areas of China, which can also obviously affect the content of the bioactive components (Yuan et al., 2012; Li et al., 2013; Tan et al., 2016). Previous investigations revealed that 87 EST-SSRs were significantly different between *L. japonica* and *L. japonica* var. *chinensis* in diverse production areas (Jiang et al., 2012). Moreover, random amplified polymorphic DNA (RAPD) markers could identify five varieties of *L. japonica* collected from different geographic locations of China (Fu et al., 2013). However, only limited information is available on the biosynthetic enzymes involved in the synthesis of active compounds and their regulation in different organs and varieties of *L. japonica* collected from diverse production areas. Therefore, it is necessary to focus on elucidating the molecular regulatory mechanisms of secondary metabolism in flower buds, stems, and leaves from both varieties of *L. japonica* in various production areas.

MicroRNAs (miRNAs), which are approximately 19 to 24 nucleotides (nt) in length, are a class of small endogenous non-coding RNAs (sRNA) that originate from their precursors (short stem-loop structures) within primary transcripts (pri-miRNA; Bartel, 2004; Barakat et al., 2007). The mature miRNAs play a key role in negative control of gene expression mainly by inhibiting the translation of target genes or by cleaving their target mRNAs, and thereby exert control over a series of key developmental and stress resistance processes (Lu et al., 2008; Bartel, 2009). To date, 8496 mature miRNAs and 6992 precursor miRNAs (pre-miRNAs) have been identified in Viridiplantae (miRBase ^1^, April 5, 2016), among which six frequently used medicinal herbs have been investigated, including *Panax ginseng, Digitalis purpurea, Rehmannia glutinosa, Salvia sclarea, Citrus reticulate*, and *C. sinensis* (Supplementary Table S1). Increasing evidence indicates that miRNAs play important regulatory roles in diverse biological and metabolic processes such as plant secondary metabolism and environmental stress resistance (Shi et al., 2010; Wu et al., 2012a ; Carlile and Werner, 2014). Thus, miRNAs have the potential to serve as biomarkers for differences in medicinal plant varieties and cultivation environmental conditions. Moreover, this class of critical regulators could be a useful tool to determine the regulation mechanisms of secondary metabolism in both varieties of *L. japonica* in response to multiple environmental factors. However, only limited honeysuckle miRNAs have been reported to date (Zhou et al., 2014; Hirschi et al., 2015; Yang et al., 2015).

In miRNA expression studies, the most common choices of reference genes are ribosomal RNAs (e.g., 5S RNA) and small nuclear RNAs (e.g., U6; Reid et al., 2006; Lagesen et al., 2007). However, the expression levels of these genes may vary across different samples or because of external factors (Kou et al., 2012; Wang et al., 2014; Wu et al., 2014). Moreover, a commonly used reference gene in an organism may not be appropriate in other organisms or under different conditions. Thus, it will be necessary to evaluate the possibility that endogenous miRNAs in plant tissues may serve as potential internal reference genes. In recent years, there have been extensive reports on the expression of miRNAs. For example, mi167 and mi159 are the two most suitable reference genes in wheat (Hao et al., 2012), and miR169, miR171/170, and miR172 are stably expressed in lettuce under abiotic stresses (Borowski et al., 2014). In addition, the combination of miR156b and miR1520d was reported as a normalizer of soybean miRNA rather than the more commonly used protein-coding genes (Kulcheski et al., 2010). However, to our knowledge, no systematic study has been carried out in different organs and varieties of *L. japonica* for validation of reference miRNAs.

Here, we evaluated the stability of a set of 16 candidate reference genes, including two general reference genes (u437272 from the 5S ribosomal RNA gene and u1325500 from the U6 small nuclear RNA gene), to identify the most suitable reference miRNA for qRT-PCR data normalization in different tissues and varieties of *L. japonica* obtained from different production areas. The optimum pairs of internal control genes were identified using GeNorm, NormFinder, and BestKeeper statistical algorithms. Our results provide an important basis for further studies on molecular regulatory mechanisms of secondary metabolism in flower buds, leaves, and stems and investigation of the potential biomarkers for differences in varieties of *L. japonica*.

## Materials and Methods

### Honeysuckle Sample Preparation

Honeysuckle samples from different geographic locations were collected from April to September 2013 (**Table 1**). Flower buds, young leaves, and stems were sampled before flowering for gene expression analysis. All samples were immediately frozen in liquid nitrogen and stored at -80°C. Three cultivars of honeysuckle (BJ-YTH, SD-YTH, and SD-DMH) were used for sequencing of small RNAs and characterization by RNA-seq. Each sample tissue of all honeysuckle cultivars consisted of four plants for the expression stability analysis and ranking of candidate reference genes. In addition, eight samples of each honeysuckle cultivar were used to validate the reference miRNAs. All the experiments were repeated in triplicate.

**Table 1:** The collection of honeysuckle samples in China.

Varieties	Sample symbol	Location	GPS^c^ Information
FLJ^a^	AH-SXYZ	Guangde, Anhui Province	31°03′04.4″ *N*, 119°22′26.0″ *E*, 108 *M*
	BJ-YTLB	Doudian, Beijing Province	39°37′46.3″ *N*, 116°01′37.5″ *E*, 20.9 *M*
	CQ-SDYZ	Yusheng, Chongqing Province	30°23′37.0″ *N*, 107°20′48.0″ *E*, 105 *M*
	CQ-SXYZ	Dianjiang, Chongqing Province	30°19′45.6″ *N*, 107°19′50.1″ *E*, 418.2 *M*
	FQ-DMH	Fengqiu, Henan Province	35°00′27.0″ *N*, 114°29′06.6″ *E*, 71.8 *M*
	GS-YT	Liangzhou, Gansu Province	37°53′25.5″ *N*, 102°44′47.0″ *E*, 1528 *M*
	GX-SDYZ	Leye, Guangxi Province	24°36′47.7″ *N*, 106°31′03.3″ *E*, 1300 *M*
	HB-DMH	Julu, Hebei province	37°09′33.8″ *N*, 115°05′37.3″ *E*, 30 *M*
	HN-DMH	Jianshan, Henan Province	34°36′30.3″ *N*, 113°17′12.2″ *E*, 551 *M*
	HUB-HBYZ	Luotian, Hubei province	30°51′19.9″ *N*, 115°12′17.4″ *E*, 61.5 *M*
	JS-YT	Lianyungang, Jiangsu Province	34°39′25.8″ *N*, 118°32′04.3″ *E*, 110 *M*
	NX-SDYZ	Chengershan, Ningxia Province	36°04′30.1″ *N*, 106°18′48.3″ *E*, 1896 *M*
	SD-YTLZ	Junan, Shandong Province	35°19′07.8″ *N*, 118°57′36.9″ *E*, 273 *M*
	SD-DMH	Linyi, Shandong Province	35°05′58.1″ *N*, 118°15′29.2″ *E*, 84.8 *M*
	SX-JHSH	Yangling, Shaanxi Province	34°18′14.5″ *N*, 108°05′18.2″ *E*, 500 *M*
	YN-YT	Kunming, Yunnan Province	25°11′01.8″ *N*, 102°58′50.6″ *E*, 1993 *M*
rFLJ^b^	BJ-YTH	Doudian, Beijing Province	39°37′46.3″ *N*, 116°01′37.5″ *E*, 20.9 *M*
	GS-YTH	Liangzhou, Gansu Province	37°53′25.5″ *N*, 102°44′47.0″ *E*, 1528 *M*
	HB-HYH	Julu, Hebei province	37°09′33.8″ *N*, 115°05′37.3″ *E*, 30 *M*
	JS-HYH	Lianyungang, Jiangsu Province	34°39′25.8″ *N*, 118°32′04.3″ *E*, 110 *M*
	SD-YTH	Linyi, Shandong Province	35°19′07.8″ *N*, 118°57′36.9″ *E*, 273 *M*

*^a^FLJ, *Lonicera japonica* Thunb.; ^b^rFLJ, *L. japonica* Thunb. *var.* chinensis (Watts); ^c^*global positioning system.**

### Extraction of miRNA, cDNA Preparation, and qRT-PCR

Total RNA was isolated from different tissues of *L. japonica* using a TRIzol kit (Invitrogen, Carlsbad, CA, USA) according to the manufacturer’s instructions. Isolated RNA integrity was tested by agarose gel electrophoresis. The quality and concentration of the extracted RNA were evaluated using a NanoDrop 2000 spectrophotometer (Thermo Scientific, San Jose, CA, USA).

Total RNA was thawed on ice, and miRNA was extracted using a miRcute miRNA Isolation Kit (TIANGEN, Beijing, China) following the manufacturer’s instructions. The quantity and purity of extracted miRNA were estimated by examining both the absorbance at 260 nm and the 260/280 nm ratio. Subsequently, 0.5 μg of RNA per sample were reverse-transcribed to cDNA with a miRcute miRNA First-Strand cDNA Synthesis Kit (TIANGEN, Beijing, China). The above cDNA was then diluted 1: 10 in water, after which 2 μl was used for quantitative PCR, using the miRcute miRNA qPCR Detection kit (TIANGEN, Beijing, China), performed on an ABI 7500 real-time quantitative PCR instrument (Applied Biosystems, Carlsbad, CA, USA). Primers for mature reference miRNAs were obtained from Sangon (Sangon Biotech, Shanghai, China). The reaction was performed at 94°C for 2 min, followed by 40 cycles of 94°C for 20 s, and 60°C for 34 s. Melting curves were performed immediately after the completion of the qRT-PCR and fluorescence was measured from 55 to 95°C. PCR efficiency (E; Tichopad et al., 2003) for each miRNA was determined (**Table 2**).

**Table 2:** Details of candidate reference genes and primers used in this study.

miRNA name	Accession number	miRNA ortholog^a^	Blastn *E*-value^a^	Reference	Primer Sequence (5′–3′)	PCR efficiency^b^	*R*^2^
lj-miR167a	KX018628	bna-miR167a	7e-04	Zhang et al., 2005, 2006	TGAAGCTGCCAGCATGATCT	1.034	0.999
lj-miR171b	KX018629	zma-miR171b	4e-20	Zhang et al., 2009	GTTCAGCCGAGCCAATATCAC	1.069	0.989
u30297	KX018630	mtr-miR2677	0.11	Lelandais-Brière et al., 2009	GGACTGGAATATGAACTTTGCACC	0.997	0.987
u1846379	KX018631	bra-miR9562	0.52	Jiang et al., 2014	GCCACTACGATATGAACTTTGCACT	0.959	0.999
u1760353	KX018632	gra-miR8709b	0.025	Xue et al., 2013	TTTCAATCATGTCGTTAACCCACT	0.986	0.993
u3464767	KX018633	pvu-miR319c	0.36	Arenas-Huertero et al., 2009	ATCCGAGACCTTGTAGAACCTGAC	0.901	0.996
u312335	KX018634	mtr-miR169g	0.009	Zhai et al., 2011	GCACCCTCTGGACAGCAACC	0.919	0.997
u4339213	KX018635	ath-miR169l	1.6	Xie et al., 2005; Moldovan et al., 2010	GTATCTGACCCGAAATTGACCC	0.911	0.993
u3817076	KX018636	bna-miR6035	0.091	Zhao et al., 2012	TATGGACTGCGATATGAACTTTGC	0.991	0.997
u2100564	KX018637	mtr-miR2620	0.025	Lelandais-Brière et al., 2009	TGGACTCGAATATGAACTTTGCAC	1.030	0.997
u821189	KX018638	mtr-miR5227	0.76	Devers et al., 2011	GCATTTAGCACCCCCTGGAC	0.998	0.999
u534122	KX018639	bna-miR6035	0.091	Zhao et al., 2012	TATGGACTGCGATATGAACTTTGC	1.045	0.998
u3868172	KX018640	atr-miR2950	0.094	Amborella Genome Project, 2013	GCATTTAGCACCCCCTGGAC	0.987	0.997
u4631289	KX018641	dpr-miR396	0.11	Wu et al., 2012a	CGAATGTACAACTCACTAATGCACC	0.915	0.999
u1325500	KX018642	-	-	-	CGATGGAACAGACCGAAGAATA	0.945	0.986
u437272	KX018643	-	-	-	CTGGGAAGTCCTCGTGTTG	0.982	0.995

*^a^miRBase was used to search for homologs of microRNA precursor sequences. ^b^The efficiency test for each miRNA was conducted using miRNA plasmid templates in a range 0.1–1000 pg.*

### Selection of Candidate Reference Genes and Primer Design

Using high-throughput sequencing of the miRNA transcriptome of *L. japonica*, we identified a set of fourteen miRNAs that were equally abundant in three small RNA-seq libraries (Accession number SRR3567675; Supplementary Table S2). The *Arabidopsis thaliana* 5S rRNA gene (AJ307353.2) and *Solanum tuberosum* U6 small nuclear RNA gene (Z17301.1) were used as queries in Blast searches of the NCBI nucleic acid database to search for homologous sequences in the *L. japonica* miRNAs database, using identification standards of *e*-value ≤1e-15, score ≥100. u437272 from the 5S ribosomal RNA gene and u1325500 from the U6 small nuclear RNA gene were selected to serve as general reference genes. Specific poly(A) primers were designed for miRNA cDNA synthesis as previously described (Rui and Chiang, 2005). miRNAs were polyadenylated and reverse-transcribed with a poly(A) adapter into cDNAs for real-time PCR using the miRNA-specific forward primer and the sequence complementary to the poly(A) adapter as the reverse primer. Specific forward primers for each full miRNA sequence were designed (**Table 2**) and the reverse primer sequence was universal (TIANGEN, Beijing, China) for qRT-PCR amplifications.

### Analysis of Stability of Reference miRNAs

To assess the stability of candidate reference miRNAs, statistical algorithms including GeNorm, NormFinder, and BestKeeper were utilized. The statistical software package GeNorm is based on pair-wise comparisons and calculates the *M*-value of all candidate genes (Vandesompele et al., 2002). A valuable feature provided by GeNorm output is the *V*-value that reflects the minimal number of genes required for a robust normalization factor (NF; Teste et al., 2009). NormFinder is an ANOVA-based model that provides direct measures of intra- and inter-group variation in the expression of each gene (Tong et al., 2009; Liu et al., 2012). BestKeeper determines the “optimal” housekeeping genes using pair-wise correlation analysis of all pairs of candidate genes, as well as the target gene (Pfaﬄ et al., 2004; Takle et al., 2007). Data analyses were performed with SPSS 19.0 statistical software (SPSS Inc., Chicago, IL, USA). Significance in relative expression levels of miRNAs were determined by one-way ANOVA. *P*-values below 0.05 were considered statistically significant for all tests.

## Results

### Selection of Candidate Reference Genes, Primer Specificity, and Amplification Efficiency in *L. japonica*

**FIGURE 1:**
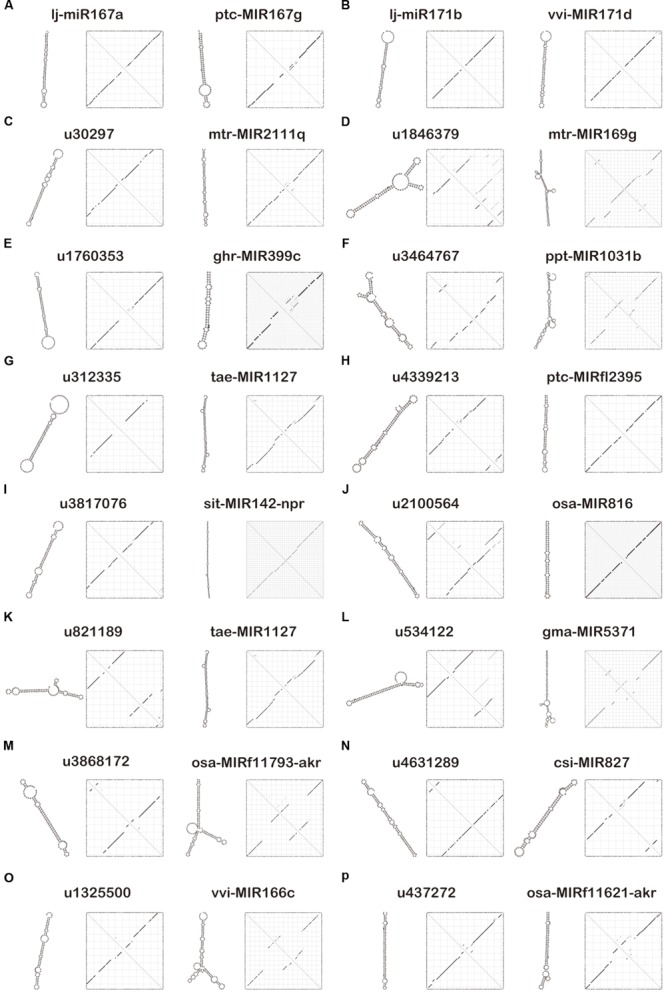
**Secondary structure prediction of 16 candidate reference miRNAs and their similar sequences using the plant microRNA database (PMRD). (A)** Secondary structures of lj-miR167a and its similar sequence ptc-MIR167g. **(B)** Secondary structures of lj-miR171b and its similar sequence vvi-MIR171d. **(C)** Secondary structures of u30297 and its similar sequence mtr-MIR2111q. **(D)** Secondary structures of u1846379 and its similar sequence mtr-MIR169g. **(E)** Secondary structures of u1760353 and its similar sequence ghr-MIR399c. **(F)** Secondary structures of u3464767 and its similar sequence ppt-MIR1031b. **(G)** Secondary structures of u312335 and its similar sequence tae-MIR1127. **(H)** Secondary structures of u4339213 and its similar sequence ptc-MIRfl2395. **(I)** Secondary structures of u3817076 and its similar sequence sit-MIR142-npr. **(J)** Secondary structures of u2100564 and its similar sequence osa-MIR816. **(K)** Secondary structures of u821189 and its similar sequence tae-MIR1127. **(L)** Secondary structures of u534122 and its similar sequence gma-MIR5371. **(M)** Secondary structures of u3868172 and its similar sequence osa-MIRf11793-akr. **(N)** Secondary structures of u4631289 and its similar sequence csi-MIR827. **(O)** Secondary structures of u1325500 and its similar sequence vvi-MIR166c. **(P)** Secondary structures of u437272 and its similar sequence osa-MIRf11621-akr.

For the evaluation of potential endogenous normalizers in *L. japonica*, 16 candidate reference miRNAs were selected from small RNA libraries, which were chosen in preference according to the following criteria: the mean variation (RPKM value) in miRNA expression levels was <1.1-fold in three miRNA sequencing libraries of different varieties of *L. japonica* and no significant differences in miRNA expression levels (*P* > 0.05). Those precursors of selected miRNAs were compared with miRBase miRNA sequences^2^ to search for homologs of microRNA sequences (**Table 2**). Additionally, a plant microRNA database (PMRD) was used to predict the secondary structures and examine sequence similarity of the selected reference miRNAs in this study (**Figure 1**). As expected, lj-miR167a and lj-miR171b were the orthologous sequences of miR167a and miR171b, which have been described in the literature as reference miRNAs in *Lotus japonicas* and maize (Zhang et al., 2009; De Luis et al., 2012). Interestingly, except for lj-miR167a and lj-miR171b, orthologous sequences of selected miRNAs were quite different but had similar secondary structure sequences, which suggested that the selected reference genes in this study might have unknown functions deserving further investigation.

The performance of each primer pair was tested by qRT-PCR. The amplification efficiencies for the 16 primers ranged from 90.1 to 106.9%, and *R*^2^ ranged from 0.986 to 0.999, according to the slopes of the standard curves (**Table 2**; **Supplementary Figure S2**). Melting curve analysis confirmed the presence of a single PCR product with a single peak from all samples, indicating that all the primers used are highly specific and efficient for qRT-PCR amplification (**Supplementary Figure S3**).

### Expression Profiling of Candidate Reference miRNAs

**FIGURE 2:**
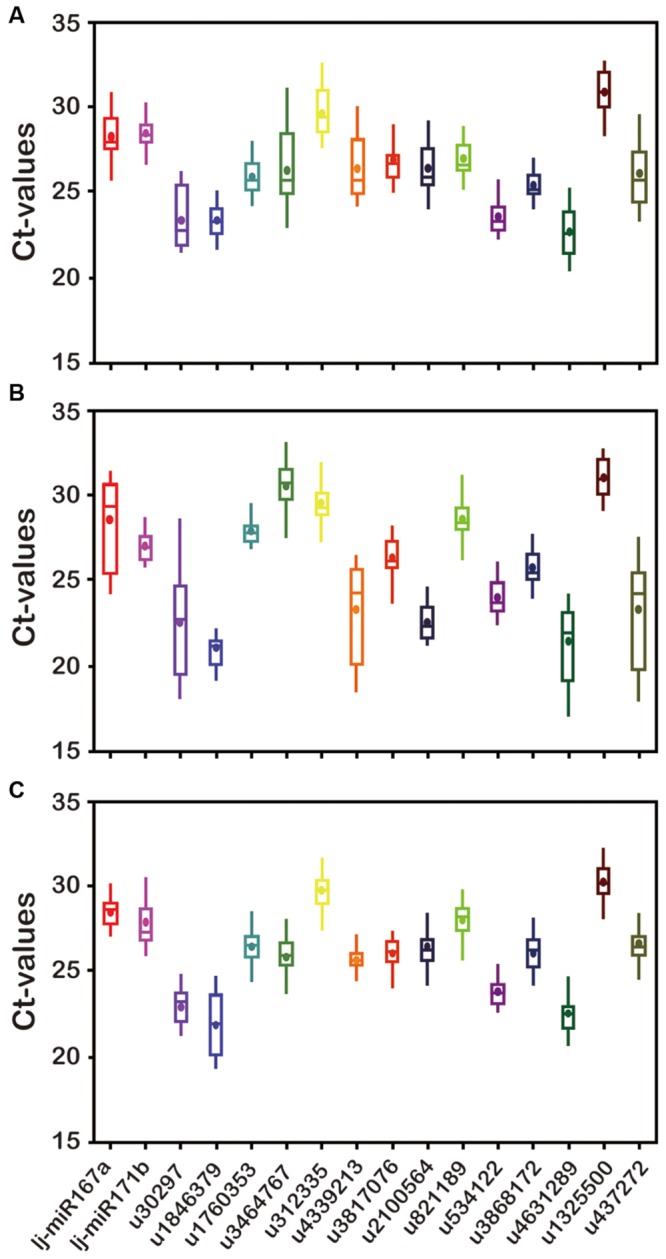
**Transcript abundance of 16 candidate genes analyzed by GeNorm in three *Lonicera japonica* datasets. (A)** Flower buds; **(B)** leaves; **(C)** stem. The solid line represents the median value and the boxes are 25th and 75th percentiles. The average is indicated by the point in the box. Whisker caps represent the maximum and minimum *C*_t_ values.

Expression levels of all 16 candidate reference genes were determined by qRT-PCR. The *C*_t_ values showed differential transcript levels in three tissue samples examined, with lower *C*_t_ values suggesting higher transcript abundance and vice versa. The range of *C*_t_ values indicated the stability of reference gene expression; the wider the range of *C*_t_ values for a gene, the more unstable the gene’s expression (Hong et al., 2008; Gao et al., 2012). As shown in **Figure 2**, the mean *C*_t_ value of all genes ranged from 21 to 31. Among all the genes, u1846379 and u4631289 were the two most abundant with mean *C*_t_ values <24, and u1325500 always had the lowest level of expression with mean *C*_t_ value >30, irrespective of the samples analyzed. The *C*_t_ value could change in different tissues. In general, u2100564 accumulated in leaves far more than in other tissues (*P* < 0.05); however, u3464767 had a lower level of expression in leaves compared with stem and flower buds (*P* < 0.05). In addition, the range of *C*_t_ values for u30297, u3464767, and u4339213 was above six cycles in flower buds, a range wider than that of the other genes tested, which indicated these miRNAs were unstable and inappropriate for reference genes. Notably, u3817076, u821189, u534122, and u3868172 showed a comparatively stable and narrower range of expression variation in all sample sets, indicating that they could possibly serve as reliable reference genes. In contrast, Ct values of u30297, u4339213, and u437272 were highly variable in all sample sets. This variation may be mainly the result of the different expression levels of these miRNAs in different tissues or the diversity in RNA yield and quality of different samples.

### Expression Stability Analysis and Ranking of Candidate Reference miRNAs

**FIGURE 3:**
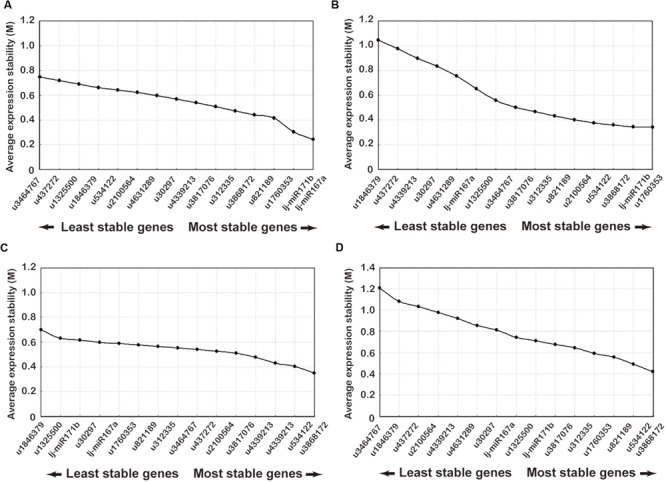
**Ranking of candidate reference genes using GeNorm analysis of qRT-PCR of candidate reference genes.** The *x*-axis from left to right indicates the ranking of the genes according to their expression stability: **(A)** flower buds; **(B)** leaves; **(C)** stems; **(D)** all samples.

Expression stability of the candidate reference genes was determined by GeNorm, NormFinder, and RefFinder which evaluated the stability ranking of each reference gene using the *C*_t_ values across all the experimental sets and tissue samples (Kozera and Rapacz, 2013; Luo et al., 2014) (**Figure 3**; Supplementary Tables S3 and S4). The stability ranking analyses done by each of the three software packages are detailed below.

**FIGURE 4:**
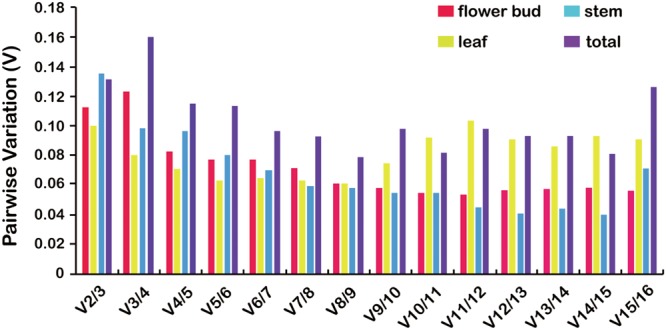
**Pairwise variation (V) analysis to determination the optimal number of reference genes for normalization using GeNorm analysis.** GeNorm calculates pairwise variation (*V*_n_/*V*_n+1_) for the normalization factors NF_n_ and NF_n+1_ to determine (*V* < 0.15) the optimal number of reference genes.

The GeNorm software, which has been used for reference gene selection in many plants, was developed for ranking the candidate genes and determination of the number of reference gene that should be applied. This method is based on the principle that the expression ratio of two ideal internal reference genes is identical in all samples regardless of the cell types or treatments. The pairwise variation of a particular gene with all the other reference genes is calculated and defined as the *M*-value, where a lower *M*-value represents a slight variation. The gene expression stability of all 16 candidate reference genes was evaluated using the GeNorm statistical algorithm. According to GeNorm analysis, the cut-off range of the stability value (*M*) is <1.5, so the gene with the lowest *M*-value is considered to be the most stable reference gene in terms of gene expression and vice versa. All tested datasets reached a high expression stability with relatively low *M*-values far below the default limit of *M* < 1.5. Gene expression stability of the 16 candidate genes was analyzed on four different datasets: (1) the *M*-values of lj-mir167a (*M* = 0.21) and lj-mir171b (*M* = 0.25) were the least followed by u1760353 and u821189 in flower buds (**Figure 3A**); (2) u1760353 (*M* = 0.31) and lj-mir171b (*M* = 0.39) were the most stable miRNAs in leaves followed by u3868172 and u534122 (**Figure 3B**); (3) u534122 (*M* = 0.32) and u3868172 (*M* = 0.41) were the most stable miRNAs in stem samples followed by u4339213 and u3817076 (**Figure 3C**); (4) the gene pair u534122 and u3868172 was the most stable combination of genes in all tissue datasets (**Figure 3D**). Although the *M*-values of lj-mir167a and lj-mir171b were the least in flower buds, they were not as stable in stems and leaves as u534122 and u3868172. Taking all of the above datasets together, u534122 and u3868172 ranked as the highest stable reference genes, indicating that they have the potential to be widely used for multiple purposes.

The GeNorm algorithm was also used to determine the optimal number of reference genes, as inclusion of two or more internal control genes for transcript normalization in qRT-PCR experiments is considered better for obtaining precise and consistent results. GeNorm determines the pairwise variation (*V*_n_/*V*_n+1_) between sequential NF (NF_n_ and NF_n+1_) in each sample set. A threshold of 0.15 was proposed to indicate that inclusion of an additional reference gene is unnecessary. Thus, according to the stability value plot (**Figure 4**), the top “*n*” reference genes are sufficient as internal controls. Taking all tissue samples together, the *V*_2/3_ values were 0.1134, 0.1012, 0.1362, and 0.1322 using four different statistical algorithms, respectively (**Figure 4**), indicating that the top two reference miRNAs would be adequate for normalization, with a third miRNA being unnecessary. Thus, u534122 and u3868172 should be included as reference genes for accurate gene expression normalization in different tissues of *L. japonica*.

NormFinder is a model-based algorithm to estimate expression variation of candidate reference genes. This strategy enables estimation of not only the overall variation of the genes but also the variation among subgroups of the sample set. This program ranked the candidate miRNAs based on intra- and inter-group variation, with a lower stability value indicating more stable expression. As shown in Supplementary Table S3, NormFinder ranked the 16 candidate miRNAs in all samples from lowest to highest stability values as follows: u3868172, u534122, u312335, lj-mir171b, lj-mir167a, u3817076, u821189, u1760353, u4631289, and u30297. Similar to the results from GeNorm, NormFinder also identified u534122 and u3868172 as the most stably expressed miRNAs in all samples, with u3464767 being the least stably expressed.

BestKeeper analyzes gene expression variation for candidate genes by calculating standard deviation (SD) and a pairwise correlation, and the lowest SD value indicates the most stable reference miRNA expression. As shown in Supplementary Table S4, u534122 and u3868172 were the most stably expressed genes with the lowest SDs in all samples (0.77 and 0.84, respectively). Thus, the results obtained through the BestKeeper program were consistent with those obtained through the GeNorm and NormFinder analyses.

Three ordered lists were generated according to the corresponding statistical values: *M*-value by GeNorm, stability value by NormFinder, and the correlation coefficient value of BestKeeper. According to the above results, u534122 and u3868172 represent the most adequate normalization candidate miRNAs tested. Only small differences between GeNorm and NormFinder were found. The combination of lj-miR171b and u1760353 was most stable in leaves using GeNorm, while lj-miR171b and u2100564 were the first and second most stable using NormFinder, respectively. In stem samples, the combination of u534122 and u3868172 was most stable using GeNorm, while u534122 and u4339213 were the first and second most stable using NormFinder, respectively. However, our result demonstrated that, in general, u534122 and u3868172 are the two most reliable reference genes.

### Validation and Analysis of Candidate Reference miRNAs

**FIGURE 5:**
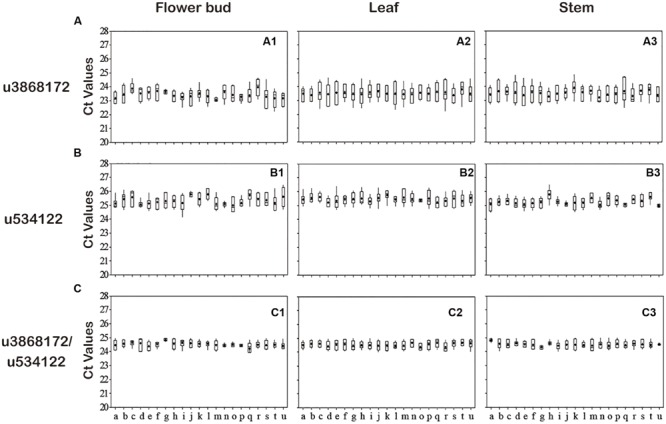
**Expression levels of selected candidate reference miRNAs among different varieties.** qRT-PCR analysis was conducted in *L. japonica* derived from different varieties in different production areas (*n* = 8). **(A)** Expression levels of u3868172 among different varieties. **(B)** Expression levels of u534122 among different varieties. **(C)** Expression levels of u3868172/u534122 among different varieties. Values are given as real-time PCR cycle threshold numbers (*C*_t_ values). The solid line represents the median value, and the boxes are 25th and 75th percentiles. The average is indicated by the point in the box. Whisker caps represent the maximum and minimum *C*_t_ values. (a = HN-DMH, b = FQ-DMH, c = CQ-SDYZ, d = SD-YTLZ, e = SD-DMH, f = GX-SDYZ, g = AH-SXYZ, h = HUB-HBYZ, i = CQ-SXYZ, j = SX-JHSH, k = NX-SDYZ, l = HB-DMH, m = YN-YT, n = JS-YT, o = BJ-YTLB, p = GS-YT, q = JS-HYH, r = BJ-YTH, s = HB-HYH, t = SD-YTH, u = GS-YTH).

**FIGURE 6:**
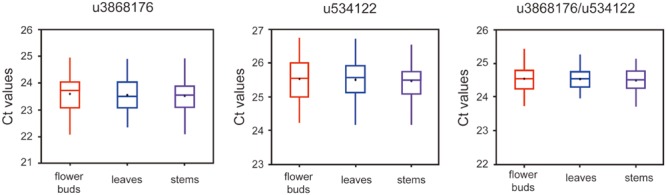
**Expression levels of selected candidate reference miRNAs among different tissues.** qRT-PCR analysis was conducted in *L. aponica* derived from all samples (*n* = 21 × 8).

To validate further the stability of u3868172 and u534122, we investigated their expression levels in different varieties of *L. japonica* (*n* = 8) collected from 21 geographic locations of China. This collection was also the basis for studying regulation mechanisms of secondary metabolism in both varieties of *L. japonica* in response to multiple environmental factors (**Table 2**). The expression levels of u3868172 and u534122 were also analyzed in three organs of these samples. The *C*_t_ value variance ranges of u3868172 were 1.33, 1.26, and 1.45 in flower buds, leaves, and stems (**Figure 5A**), respectively. Similarly, u534122 also showed relatively stable expression with *C*_t_ value variance ranges of 1.57 in flower buds, 1.62 in leaves, and 1.83 in stems (**Figure 5B**). Furthermore, the least expression variation in the different samples was shown by the combination of u3868172 and u534122, with *C*_t_ variance value ranges of 0.862 in flower buds, 0.878 in leaves, and 0.865 in stems (**Figure 5C**). In addition, within different samples, u3868172/u534122 had standard deviations of less than 1 (**Figure 5C**). Moreover, the expression levels of u3868172 and u534122 were also analyzed in three organs in all samples to validate their usage in miRNA regulation in these tissues (**Figure 6**). The *C*_t_ value variance range of u3868172/u534122 was the narrowest among the candidate miRNAs (**Figures 5** and **6**), which indicated that the combination u3868172/u534122 could be recommended as the optimal reference for miRNAs in *L. japonica*.

## Discussion

The quality of herbal medicine has been very difficult to control and to evaluate primarily because of the complexity and incomplete knowledge of the active medicinal compounds. *L. japonica*, widely used in traditional medicine, contains many active compound, whose contents differed significantly among the organs, varieties, and production areas of the herbal medicines. Thus, recent focus on variation in quality of medicinal material has been directed toward understanding the molecular regulatory mechanisms of secondary metabolism through transcriptomics or functional genomics approaches. miRNA genes are an important class of fine-tuning regulators, playing a major role in a wide range of developmental, biological, and metabolic processes in plants, including metabolism, stress response, vegetative phase changes, organogenesis, and signal transduction (Mathiyalagan et al., 2013). To date, a large number of miRNA families have been described in plants, some of which are directly associated with secondary metabolism synthesis (Ekström et al., 2015). Increasingly, miRNAs have been identified and characterized in some important medicinal herbs (Song et al., 2009; Wu et al., 2012b) and different tissues of medicinal plants (Xu et al., 2014; Khaldun et al., 2015). Further, the prediction of phenylpropanoid and terpenoid biosynthetic pathway genes targeting miRNAs in various plant species (Shi et al., 2010; Mathiyalagan et al., 2013), as well as evidence of flower development (Song et al., 2015; Niu et al., 2016) and stress resistance (Navarro et al., 2006; Lu et al., 2008; Zhang Y. et al., 2016), miRNAs produce insight into the study of miRNAs in different tissues and varieties of *L. japonica*.

qRT-PCR has emerged as a useful technique for validation of transcriptomics data owing to its precision, accuracy, convenience, speed, and sensitivity. However, accurate normalization of gene expression remains a major criterion for precise qRT-PCR analysis, as normalization helps in adjusting variation introduced at various steps of qRT-PCR arising from sample-to-sample variation, variation in RNA integrity, PCR efficiency, and cDNA sample loading (Shivhare and Lata, 2016). To date, numerous RNA species, including rRNA, snRNA, and synthetic miRNAs as spike-in controls, have been used as normalizers (Peltier and Latham, 2008). However, few studies have focused on selecting suitable reference miRNAs for different varieties and different tissues in medicinal plants. Thus, the present study performed comprehensive analysis of candidate reference genes for miRNA gene expression studies in different tissues of *L. japonica* varieties. Fourteen *L. japonica* miRNA candidate genes were selected for the comprehensive expression stability analysis from three small RNA-seq libraries. The performance of each candidate gene was tested in different samples (flower buds, leaves, and stems) to help determine suitable sets of reference genes for expression profiles studies in *L. japonica*.

In this research, we provide evidence that miRNAs could have high expression stability in medicinal plants, which has also been recently demonstrated in other plant species (Kulcheski et al., 2010; Hao et al., 2012; Borowski et al., 2014). The commonly used GeNorm, NormFinder, and BestKeeper algorithms identified the best suitable reference gene sets for each group of tissue samples in *L. japonica*. Although the results from the three algorithms did not rank genes in exactly the same order, these ranking discrepancies were previously reported and reflect differences in the calculation methods of these three approaches. u3868172 and u534122 appeared to be the two most optimal reference genes after analysis with GeNorm; a similar result was confirmed through NormFinder and BestKeeper analyses. After verification of the expression of u3868172 and u534122, the individual expressions of u3868172 and u534122 showed statistical differences within breeding groups, indicating that the selection of a single reference miRNA may be not adequate. Further, the combination of u3868172/u534122 was more suitable as the inner reference gene and was not affected by differences in breeding or plant organs. Additionally, secondary structures of u3868172 and u534122 precursors were predicted by PMRD, whose target genes could be investigated by available *L. japonica* transcriptome data in the future. Therefore, the group of miRNA reference genes in this study was validated not only as biomarkers for *L. japonica* but also for performing normalization of the expression of a new miRNAs specific to *L. japonica*.

Finally, we hope that our work will facilitate the exploration of regulatory mechanisms related to secondary metabolism for production of useful compounds in *L. japonica*.

## Author Contributions

The work presented here was carried out in collaboration between all authors. Completed the main experiment and draft the manuscript: YW and JL. Defined the research theme: YY and LH. Completed sequencing the small RNA libraries data: XW and GW. Completed the Data analysis: JZ. Completed validation of candidate reference miRNAs: SL and TC. Revised the manuscript: CJ and LZ.

## Conflict of Interest Statement

The authors declare that the research was conducted in the absence of any commercial or financial relationships that could be construed as a potential conflict of interest.

## Footnotes

1 www.mirbase.org/cgi-bin/browse.pl

2 http://www.mirbase.org/

## Supplementary Material

FIGURE S1 **Some *Lonicera japonica* samples derived from different varieties in different producing areas **(A)** FLJ in Beijing; **(B)** FLJ in Beijing; **(C)** FLJ in Jiangsu **(D)** FLJ in GS; **(E)** FLJ in SD; **(F)** FLJ in Hubei; **(G)** FLJ in Ningxia; **(H)** FLJ in Chongqing; **(I)** rFLJ in Beijing; **(J)** rFLJ in Hebei; **(K)** rFLJ in Jiangsu; **(L)** rFLJ in GS**.

FIGURE S2 **Linear correlation between *C*_t_ values and log concentration of miRNAs in a serially diluted miRNA plasmid standards.** miRNAs expression levels were measured in 10-fold serial dilutions.

FIGURE S3 **Melting curves for sixteen candidate reference genes.** Temperature is displayed in the *x*-axis, and the fluorescence signal is displayed in the *y*-axis.
