# Supplementary material for: Validation of Suitable Reference Genes for Assessing Gene Expression of MicroRNAs in Lonicera japonica
Source: Front Plant Sci. 2016 Jul 26;7:1101. doi: 10.3389/fpls.2016.01101 (PMC4961011; doi:10.3389/fpls.2016.01101)
Supplement: Supplementary file 1 [file Table_1.PDF]

Supplemental Table S1. Summary of miRNA number in viridiplantae searching in miRBase (www.mirbase.org).

	**No. of pre-mRNAs**	**No. of mature miRNAs**
Chlamydomonas reinhardtii	50	86
Cunninghamia lanceolata	4	4
Picea abies	40	41
Pinus densata	29	29
Pinus taeda	35	36
Physcomitrella patens	229	280
Selaginella moellendorffii	58	64
Amborella trichopoda	124	129
Panax ginseng	29	32
Cynara cardunculus	48	57
Helianthus annuus	6	7
Helianthus argophyllus	3	3
Helianthus ciliaris	3	3
Helianthus exilis	2	2
Helianthus paradoxus	3	3
Helianthus petiolaris	3	3
Helianthus tuberosus	16	16
Arabidopsis lyrata	205	384
Arabidopsis thaliana	325	427
Brassica napus	90	92
Brassica oleracea	10	11
Brassica rapa	96	157
Carica papaya	79	81
Cucumis melo	120	120
Hevea brasiliensis	31	31
Manihot esculenta	153	153
Ricinus communis	63	63
Acacia auriculiformis	7	7
Arachis hypogaea	23	32
Acacia mangium	3	3
Glycine max	573	639
Glycine soja	13	13
Lotus japonicus	62	67
Medicago truncatula	672	756
Phaseolus vulgaris	8	10
Vigna unguiculata	18	18
Avicennia marina	2	3
Digitalis purpurea	13	13
Rehmannia glutinosa	32	37

Salvia sclarea	18	18
Linum usitatissimum	124	124
Gossypium arboreum	1	1
Gossypium herbaceum	1	1
Gossypium hirsutum	78	80
Gossypium raimondii	296	296
Theobroma cacao	82	82
Aquilegia caerulea	45	45
Bruguiera cylindrica	4	4
Bruguiera gymnorhiza	4	4
Malus domestica	206	207
Prunus persica	180	214
Citrus clementina	5	5
Citrus reticulata	4	4
Citrus sinensis	60	64
Citrus trifoliata	6	6
Populus euphratica	4	4
Populus trichocarpa	352	401
Nicotiana tabacum	162	164
Solanum lycopersicum	77	110
Solanum tuberosum	224	343
Vitis vinifera	163	186
Aegilops tauschii	88	173
Brachypodium distachyon	317	525
Elaeis guineensis	6	6
Festuca arundinacea	15	15
Hordeum vulgare	69	71
Oryza sativa	592	713
Sorghum bicolor	205	241
Saccharum officinarum	16	16
Saccharum sp.	19	20
Triticum aestivum	116	119
Triticum turgidum	1	1
Zea mays	172	321
**Total number**	6992	8496
