# Supplementary material for: Validation of Suitable Reference Genes for Assessing Gene Expression of MicroRNAs in Lonicera japonica
Source: Front Plant Sci. 2016 Jul 26;7:1101. doi: 10.3389/fpls.2016.01101 (PMC4961011; doi:10.3389/fpls.2016.01101)
Supplement: Supplementary file 2 [file Table_2.PDF]

Supplemental Table S2. Selection of 14 candidate reference genes from three small RNA-seq libraries.

miRNA name	No. of Reads			miRNA length (bp)	miRNA sequence (5’ to 3’)
	SO1[a]	SO2[b]	SO3[c]		
lj-miR167a	404	464	444	20	UGAAGCUGCCAGCAUGAUCU
lj-miR171b	306	225	184	20	UUGAGCCGUGCCAAUAUCAC
u30297	130	118	109	24	GGACUGCAAUAUGAACUUUGCACC
u1846379	35	30	36	22	ACUACAAUAUGAACUUUGCACU
u1760353	276	195	256	24	UUUCAAUCAUGUAGUUAACCCACU
u3464767	206	145	220	24	AUCCGAGACCUUGCAGAACCUGAC
u312335	439	452	488	24	UUUAGCACCCUCUGGACAGCAACC
u4339213	182	136	144	24	GCGUGUCUGACCCGAAAUUGACCC
u3817076	142	135	141	24	UAUGGACUGCAAUAUGAACUUUGC
u2100564	337	334	298	24	UGGACUGCAAUAUGAACUUUGCAC
u821189	112	124	128	24	AAAUGCAUUUAGCACCCCCUGGAC
u534122	182	181	207	24	UAUGGACUGCAAUAUGAACUUUGC
u3868172	112	124	125	24	AAAUGCAUUUAGCACCCCCUGGAC
u4631289	192	231	201	24	GAAUGUACAACUCACUAAUGCACC

a. SO1: RNA-seq library of BJ-YTH (number of total clean reads 11057240) ;

b. SO2: RNA-seq library of SD-YTH (number of total clean reads 8673800) ;

c. SO3: RNA-seq library of SD-DMH (number of total clean reads 8743421)
