# Supplementary material for: Validation of Suitable Reference Genes for Assessing Gene Expression of MicroRNAs in Lonicera japonica
Source: Front Plant Sci. 2016 Jul 26;7:1101. doi: 10.3389/fpls.2016.01101 (PMC4961011; doi:10.3389/fpls.2016.01101)
Supplement: Supplementary file 3 [file Table_3.PDF]

Supplemental Table S3. Stability values of each reference miRNA from the NormFinder algorithm. Ranking of candidate reference genes based on stability values calculated by NormFinder.

Sort	All sample		Flower bud		leafs		Stem	
	**miRNA name**	**Stability value**	**miRNA name**	**Stability value**	**miRNA name**	**Stability value**	**miRNA name**	**Stability value**
1	u3868172	0.262	lj-mir171b	0.194	lj-mir171b	0.200	u534122	0.170
2	u534122	0.315	lj-mir167a	0.198	u3868172	0.213	u4339213	0.223
3	u312335	0.322	u1760353	0.210	u2100564	0.215	u3868172	0.261
4	lj-mir171b	0.336	u821189	0.247	u534122	0.257	u3817076	0.257
5	lj-mir167a	0.367	u312335	0.265	u1760353	0.356	u437272	0.283
6	u3817076	0.370	u3868172	0.268	u821189	0.365	u2100564	0.290
7	u821189	0.449	u4339213	0.356	u3464767	0.371	u4631289	0.286
8	u1760353	0.519	u3817076	0.362	u312335	0.431	u3464767	0.288
9	u4631289	0.523	u4631289	0.387	u3817076	0.460	u312335	0.300
10	u30297	0.526	u2100564	0.399	lj-mir167a	0.460	u30297	0.322
11	u1325500	0.543	u534122	0.417	u1325500	0.618	u821189	0.332
12	u4339213	0.726	u30297	0.424	u4631289	0.693	lj-mir171b	0.374
13	u2100564	0.787	u1846379	0.430	u30297	0.748	u1760353	0.393
14	u1846379	0.855	u1325500	0.526	u4339213	0.792	lj-mir167a	0.397
15	u437272	0.890	u437272	0.544	u437272	0.988	u1325500	0.427
16	u3464767	1.352	u3464767	0.566	u1846379	0.992	u1846379	0.767
