# Supplementary material for: Validation of Suitable Reference Genes for Assessing Gene Expression of MicroRNAs in Lonicera japonica
Source: Front Plant Sci. 2016 Jul 26;7:1101. doi: 10.3389/fpls.2016.01101 (PMC4961011; doi:10.3389/fpls.2016.01101)
Supplement: Supplementary file 4 [file Table_4.PDF]

Supplemental Table S4. Stability values of each reference miRNA from the BestKeeper algorithm. Ranking of candidate reference genes based on stability values calculated by BestKeeper.

Sort	All sample		Flower bud		leafs		Stem	
	**miRNA name**	**SD**	**miRNA name**	**SD**	**miRNA name**	**SD**	**miRNA name**	**SD**
1	u3868172	0.775	u3868172	0.671	lj-mir171b	0.676	u4339213	0.503
2	u534122	0.848	u1846379	0.755	u1760353	0.572	u534122	0.586
3	u3817076	0.859	u534122	0.779	u1846379	0.848	u3817076	0.677
4	lj-mir171b	0.918	lj-mir171b	0.781	u2100564	0.851	u821189	0.724
5	u1325500	1.017	u821189	0.829	u3868172	0.881	u3464767	0.727
6	u4631289	1.062	u1760353	0.841	u534122	0.895	u3868172	0.742
7	lj-mir167a	1.069	u1325500	0.905	u312335	0.917	u4631289	0.747
8	u821189	1.175	lj-mir167a	0.997	u1325500	0.918	u1760353	0.786
9	u1760353	1.179	u3817076	1.027	u3817076	0.927	lj-mir167a	0.814
10	u312335	1.244	u2100564	1.081	u821189	0.987	u312335	0.826
11	u30297	1.347	u4631289	1.193	u3464767	1.77	u437272	0.854
12	u4339213	1.348	u312335	1.270	u4631289	1.935	u30297	0.856
13	u437272	1.430	u30297	1.437	u30297	2.068	u2100564	0.869
14	u1846379	1.433	u4339213	1.440	lj-mir167a	2.152	u1325500	0.912
15	u2100564	1.805	u437272	1.573	u4339213	2.344	lj-mir171b	1.139
16	u3464767	3.611	u3464767	1.780	u437272	2.558	u1846379	1.627
