# Supplementary figures and images for: Validation of Suitable Reference Genes for Assessing Gene Expression of MicroRNAs in Lonicera japonica
Source: Front Plant Sci. 2016 Jul 26;7:1101. doi: 10.3389/fpls.2016.01101 (PMC4961011; doi:10.3389/fpls.2016.01101)

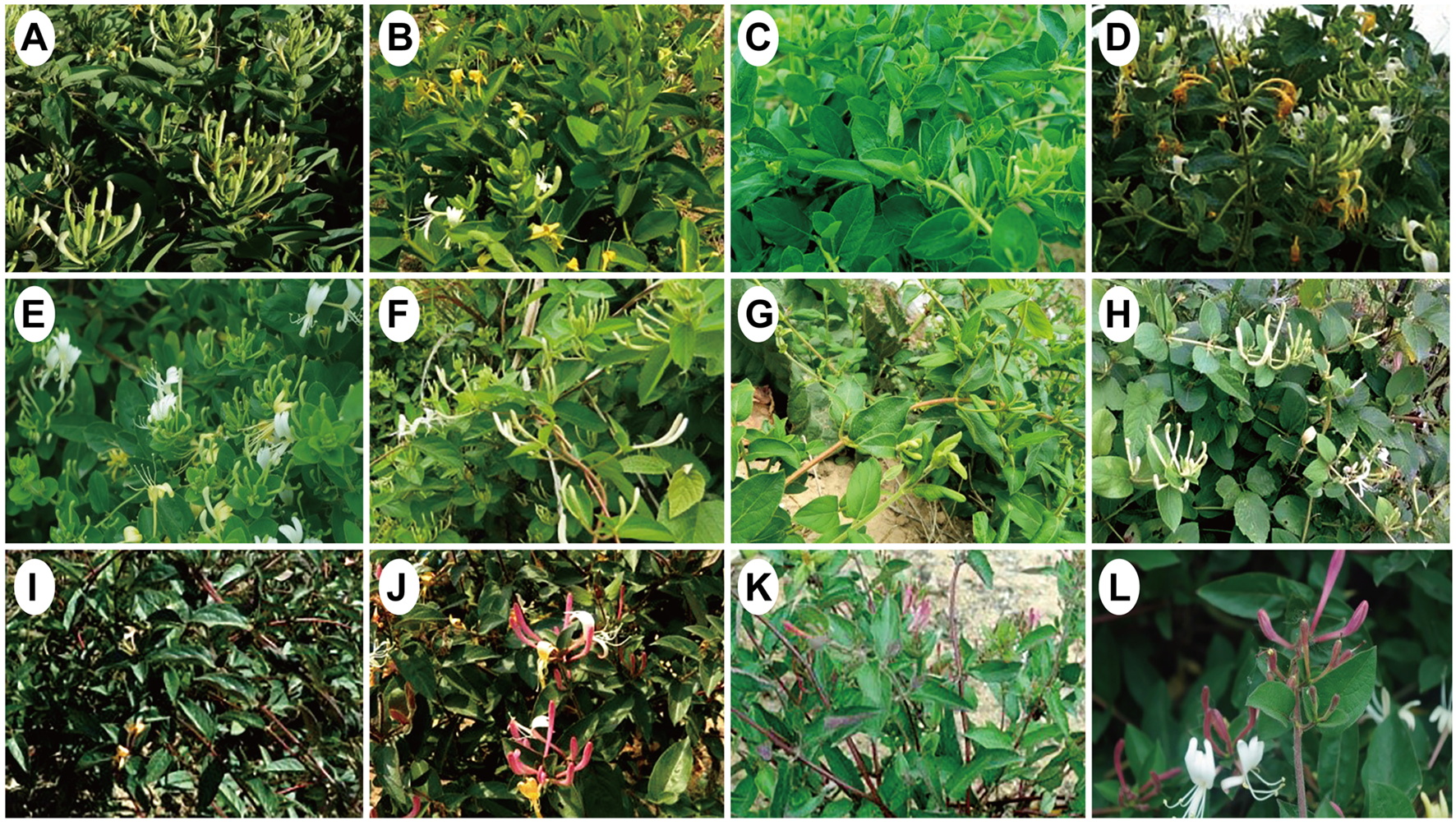

Supplement: FIGURE S1 — Some Lonicera japonica samples derived from different varieties in different producing areas (A) FLJ in Beijing; (B) FLJ in Beijing; (C) FLJ in Jiangsu (D) FLJ in GS; (E) FLJ in SD; (F) FLJ in Hubei; (G) FLJ in Ningxia; (H) FLJ in Chongqing; (I) rFLJ in Beijing; (J) rFLJ in Hebei; (K) rFLJ in Jiangsu; (L) rFLJ in GS. [file Image_1.TIF]

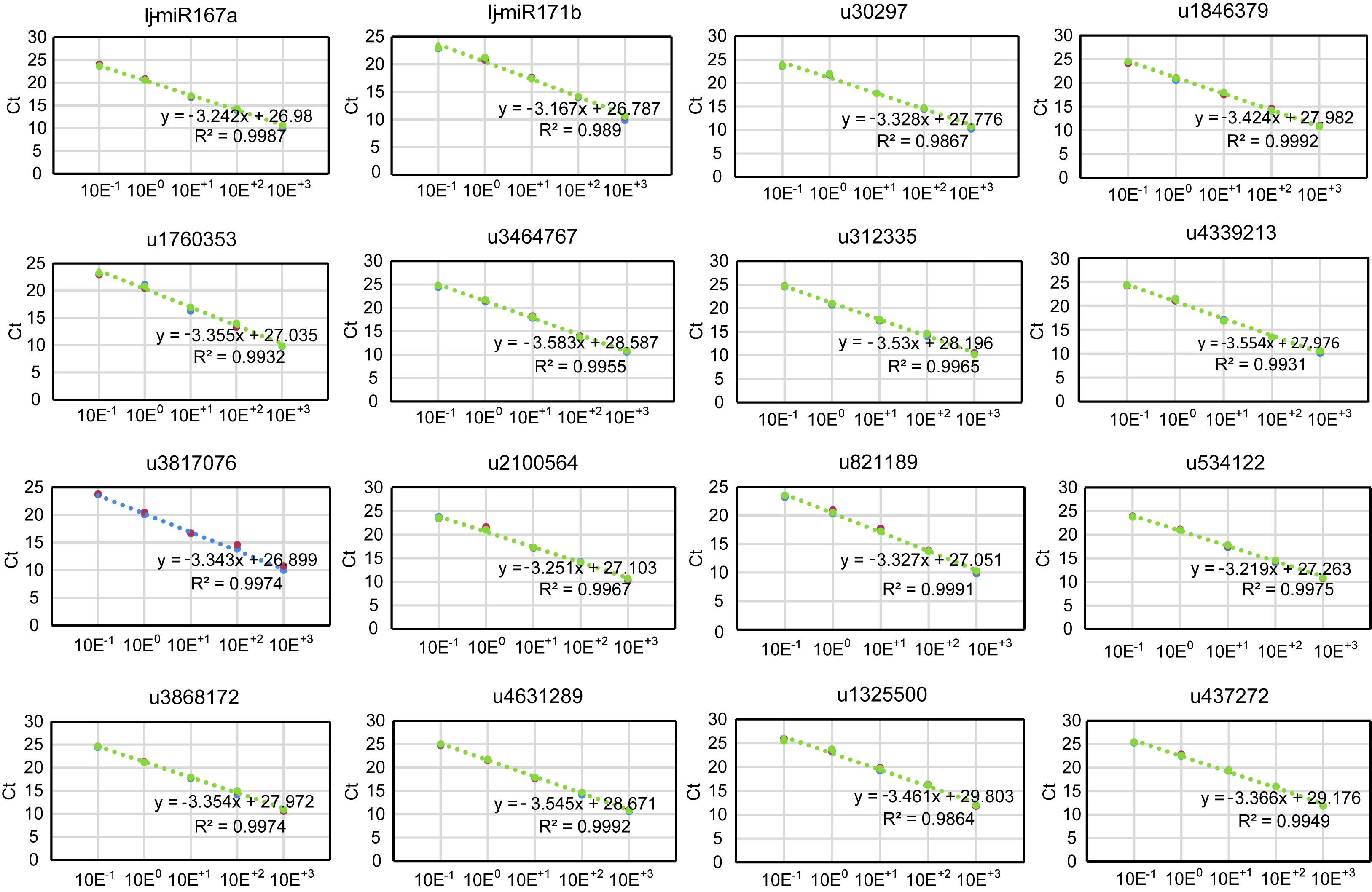

Supplement: FIGURE S2 — Linear correlation between Ct values and log concentration of miRNAs in a serially diluted miRNA plasmid standards. miRNAs expression levels were measured in 10-fold serial dilutions. [file Image_2.TIF]

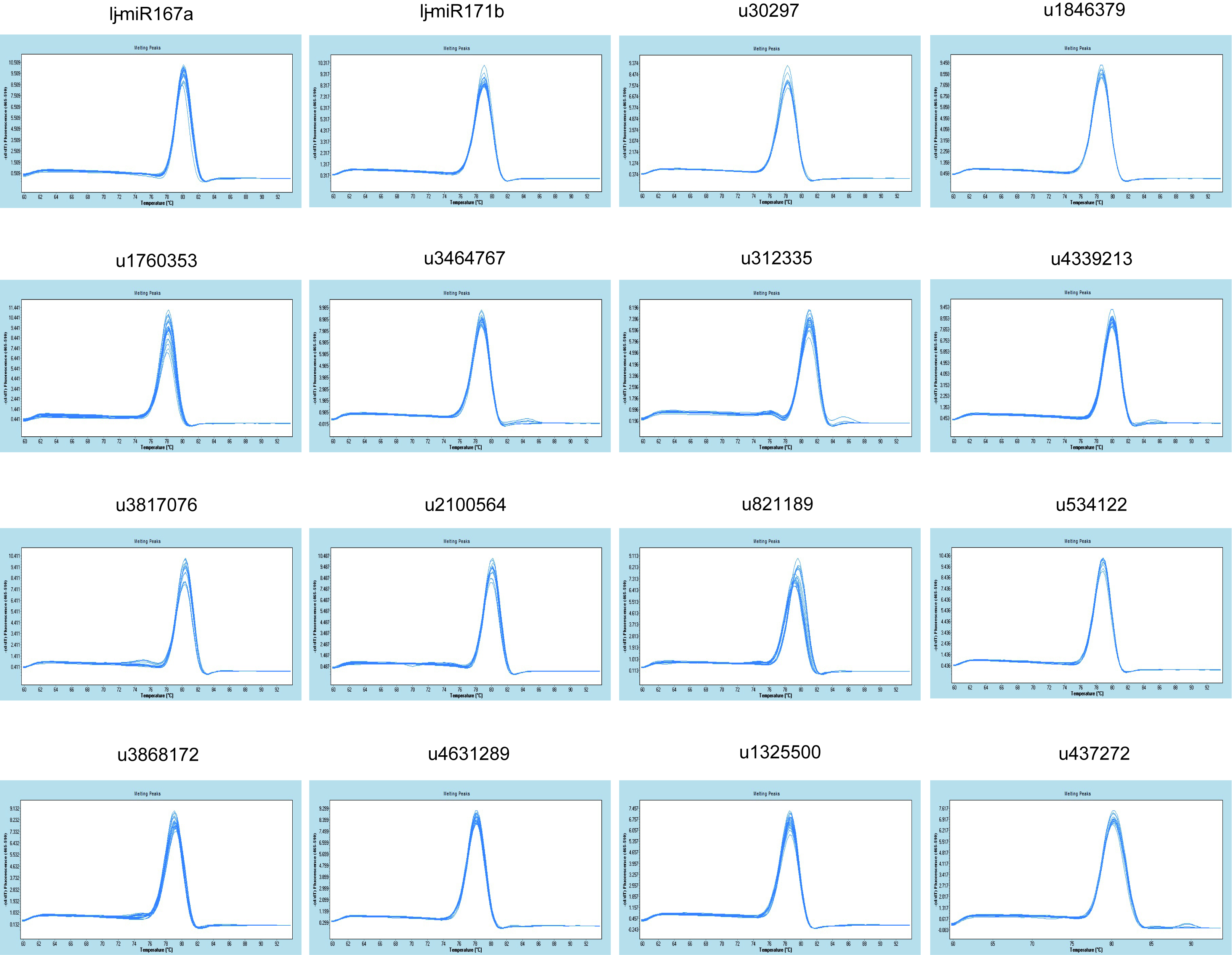

Supplement: FIGURE S3 — Melting curves for sixteen candidate reference genes. Temperature is displayed in the x-axis, and the fluorescence signal is displayed in the y-axis. [file Image_3.TIF]
